# MicroRNA Signatures in Serous Ovarian Cancer: A Comparison of Prognostic Marker Targets in African Americans and Caucasians

**DOI:** 10.3390/diseases13110360

**Published:** 2025-11-06

**Authors:** Jane M. Muinde, Celina Romi Yamauchi, Joseph Cruz, Alena A. McQuarter, Kyah Miller, Umang Sharma, Skyler Schiff, Isaac Kremsky, Saied Mirshahidi, Cody S. Carter, Salma Khan

**Affiliations:** 1 Center for Health Disparities, Loma Linda University, Loma Linda, CA 92350, USA; jmuinde@students.llu.edu (J.M.M.); ryamauchi@llu.edu (C.R.Y.); josephcruz@llu.edu (J.C.); amcquarter@students.llu.edu (A.A.M.); kyahmiller@llu.edu (K.M.); sschiff@students.llu.edu (S.S.); 2 Department of Earth and Biological Sciences, Loma Linda University, Loma Linda, CA 92350, USA; 3 School of Public Health, Loma Linda University, Loma Linda, CA 92350, USA; usharma@students.llu.edu; 4 Basic Science Research, School of Medicine, Loma Linda University, Loma Linda, CA 92350, USA; ikremsky@llu.edu; 5 Cancer Center, Biospecimen Laboratory, Loma Linda University, Loma Linda, CA 92350, USA; smirshahidi@llu.edu; 6 Department of Pathology and Human Anatomy, Loma Linda University, Loma Linda, CA 92350, USA; cocarter@llu.edu

**Keywords:** microRNAs, serous ovarian cancer, health disparities, prognostic biomarkers

## Abstract

**Simple Summary:**

Ovarian cancer (OC) is the second most common gynecologic cancer in the United States, causing more deaths than any other cancer of the female reproductive system. Mortality rates for ovarian cancer of recent African origin, compared to those of either Asian or American ancestry, have increased over the years. Identifying microRNA signatures associated with racial disparities can be an effective prognostic tool in the clinic. In this study, we identified significant genetic prognostic markers and microRNA signatures linked to them. We used both Formalin-fixed paraffin-embedded and fresh ovarian tumor tissue samples from African American and Caucasian patients with serous ovarian cancer. Total RNA extraction was performed on the tumor tissue samples, and quantitative PCR was used to determine the differential expression of various microRNAs linked to the identified prognostic markers. Determining microRNA signatures in ovarian cancer will provide information about their potential clinical relevance for future diagnosis, prognosis, and therapeutics.

**Abstract:**

**Background:** Ovarian cancer (OC) is the second most common gynecologic malignancy in the United States and remains the leading cause of death among cancers of the female reproductive system. Alarmingly, mortality rates have risen disproportionately among women of African ancestry compared to those of European or Asian descent. Identifying microRNA (miRNA) signatures that contribute to these disparities may enhance prognostic accuracy and inform personalized therapeutic strategies. **Methods:** In this study, we identified prognostic markers of overall survival in serous ovarian cancer (SOC) using data from The Cancer Genome Atlas (TCGA) and the Human Protein Atlas. Integrative bioinformatic analyses revealed three key prognostic genes—*TIMP3* (Tissue Inhibitor of Metalloproteinases-3), *BRAF* (v-raf murine sarcoma viral oncogene homolog B), and *ITGB1* (Integrin Beta-1)—as critical molecular determinants associated with survival in patients with SOC. Candidate miRNAs regulating these genes were predicted using TargetScanHuman v8.0, identifying a core regulatory set comprising miR-192, miR-30d, miR-16-5p, miR-143-3p, and miR-20a-5p. To validate their clinical relevance, formalin-fixed, paraffin-embedded (FFPE) and fresh SOC tumor samples were obtained from African American and Caucasian patients who underwent surgery at Loma Linda University (LLU) between 2010 and 2023. **Results and Discussion:** Among all these, *ITGB1* (*p* = 0.00033), *TIMP3* (*p* = 0.0035), and *BRAF* (*p* = 0.026) emerged as statistically significant predictors. Following total RNA extraction, cDNA synthesis, and quantitative reverse transcription PCR (qRT-PCR), the expression levels of these miRNAs and their target genes were quantified. In the LLU cohort, *ITGB1* and *TIMP3* were significantly upregulated in African American patients compared to Caucasian patients (*p* < 0.01 and *p* < 0.02, respectively). Among the miRNAs, miR-192-5p was particularly noteworthy, showing marginally differential expression in LLU samples (*p* = 0.0712) but strong statistical significance in the TCGA cohort (*p* = 0.00013), where elevated expression correlated with poorer overall survival (*p* = 0.021). Pathway enrichment and gene ontology analyses (miRTargetLink2.0, Enrichr) revealed interconnected regulatory networks linking miR-192, miR-16-5p, miR-143-3p, and miR-20a-5p to *ITGB1*; miR-143-3p/miR-145-5p to *BRAF*; and miR-16-5p and miR-30c/d to *TIMP3*. **Conclusions:** Collectively, these findings identify distinct miRNA–mRNA regulatory signatures—particularly the miR-192-5p–*ITGB1*/*TIMP3* axis—as potential clinically relevant biomarkers that may contribute to racial disparities and disease progression in ovarian cancer.

## 1. Introduction

Ovarian cancer (OC) remains one of the most lethal gynecologic malignancies worldwide, largely due to late-stage diagnosis and high rates of recurrence [1]. Importantly, OC represents a heterogeneous group of diseases rather than a single entity. Approximately 90% of OCs are of epithelial origin, encompassing multiple histologic subtypes—including high-grade serous, endometrioid, clear cell, and mucinous carcinomas—each defined by distinct molecular alterations, clinical behavior, and therapeutic outcomes. The remaining ~10% of OCs are non-epithelial, comprising primarily germ cell tumors and sex cord–stromal tumors. Germ cell tumors differ markedly from epithelial OCs, as they typically occur in younger women, exhibit rapid growth, and are unilateral in 95% of cases. Notably, 60–70% of germ cell tumors present at an early stage, carrying an overall favorable prognosis. These biological and clinical distinctions underscore the importance of histologic and molecular classification in guiding patient management and therapy selection [2].

Serous ovarian carcinomas (SOCs), the most prevalent form of epithelial OC, are classified by the WHO into serous cystadenomas, adenofibromas, borderline tumors, and low- and high-grade carcinomas [3]. Although OC incidence in the U.S. has declined by ~2% annually from 2017 to 2021, African American (AA) women continue to experience significantly lower 5-year survival rates compared to Caucasian American (CA) women (CDC (https://gis.cdc.gov); accessed on 20 November 2024 [4]). Population-specific genetic and molecular differences partly drive these disparities [5,6].

miRNAs are small, non-coding RNAs that regulate gene expression by degrading or inhibiting the translation of target mRNAs, thereby influencing the expression of oncogenes and tumor suppressor genes [7]. MicroRNA (miRNA) expression variations may partially explain ethnic differences in SOC, given their roles in regulating the cell cycle, apoptosis, invasion, and angiogenesis [8]. miRNAs are thus promising candidates for diagnostic and prognostic biomarkers [9,10]. Circulating miRNAs are stable in biofluids and can be detected using advanced techniques such as in situ hybridization and qRT-PCR, thereby enhancing their clinical utility [11].

Historically, High-grade SOC (HGSOC) has been associated with mutations in TP53 and BRCA1/2, which are targets of standard therapies, including platinum–taxane combinations and PARP inhibitors [12]. However, resistance and recurrence remain significant barriers. Conventional biomarkers such as CA125 and HE4 lack sensitivity and specificity, especially in early-stage disease, prompting interest in miRNA-based alternatives [13].

Several miRNAs—such as miR-200, miR-141, let-7b, and miR-199a—have been identified as prognostic in OC [14]. Genes like *ITGB1*, *TIMP3*, and *BRAF*, listed in the Human Protein Atlas (TCGA), also emerge as potential prognostic markers. *ITGB1*, often upregulated in OC, promotes tumor progression [15,16]. *TIMP3* functions as a tumor suppressor by inhibiting MMPs, promoting apoptosis, and preventing angiogenesis [17]. Downregulation of *TIMP3*—as influenced by miR-30d—is associated with poorer outcomes and increased invasiveness [18]. *BRAF* mutations, especially V600E, are linked to favorable outcomes in low-grade serous tumors [19]. MiR-143, known to target *BRAF*, has been shown to suppress tumor proliferation and chemoresistance in lung cancer models [20], suggesting similar potential in SOC.

The current study focuses on the differential expression of miR-192, miR-30d, hsa-miR-16-5p, miR-143-3p, and miR-20a-5p and their relationship with the prognostic markers such as *ITGB1*, *TIMP3*, and *BRAF*, to better understand the molecular basis of racial disparities in SOC outcomes.

## 2. Materials and Methods

### 2.1. Identification of Prognostic Markers

This study identified key genetic prognostic markers associated with overall survival in serous ovarian carcinoma (SOC) using data from The Cancer Genome Atlas (TCGA) and the Human Protein Atlas (www.proteinatlas.org) accessed on 15 January 2025. Predicted microRNAs targeting these genes were identified using TargetScanHuman v8.0, MiRTargetLink 2.0, and Enrichr (https://maayanlab.cloud/Enrichr/) accessed on 7 February 2025. For the selected miRNAs-miR-192-5p, miR-30d-5p, miR-16-1-5p, miR-143-3p, and miR-20a-5p-we analyzed differential gene expression and correlated expression profiles with overall survival among pre- and postmenopausal age groups, with staging (I to stage IV), Caucasian and African American ovarian cancer (OV) patients from the TCGA-OV cohort using the University of Alabama at Birmingham Cancer Data Analysis Portal (UALCAN).

### 2.2. Patient Cohort Information

Loma Linda University (LLU)-OV-Cohort: We conducted a cross-sectional study using patient samples that included 22 formalin-fixed, paraffin-embedded (FFPE) and 13 fresh ovarian tumor tissues, selected from African American and Caucasian cohorts. Preliminary analyses were performed on archival ovarian tumor tissues comprising 16 African American and 19 Caucasian pre- and postmenopausal women with no other concurrent malignancies. Kaplan–Meier (KM) survival analysis was performed to evaluate overall survival (OS, in months) and OS status between the two ethnic groups, as summarized in Supplementary Table S1. The analysis was conducted in R (version 4.4.2) using the specified R code to assess differences in survival distributions by ethnicity. All tissue samples were obtained from the Loma Linda University Cancer Center (LLUCC) Biospecimen Laboratory. Both archival and fresh, de-identified ovarian tumor tissues were analyzed from African American (first-generation Africans residing in the United States for several decades) and Caucasian patients, categorized according to self-identified ethnicity (Supplementary Table S1).

### 2.3. RNA Extraction from FFPE Tumor Samples

Total RNA and miRNA were extracted from nineteen 10 μm thick FFPE ovarian tumor sections using the AllPrep DNA/RNA FFPE Kit (QIAGEN, Valencia, CA, USA; Catalog No. 80234) following the manufacturer’s protocol [21]. RNA concentration and purity were assessed using a NanoDrop spectrophotometer (NanoDrop Technologies, Waltham, MA, USA). Samples with an OD 260/280 ratio below 1.8 were excluded from further analysis. Reverse transcription was performed using the Mir-X™ First Strand Synthesis Kit (TaKaRa Bio, 2560 Orchard Pkwy, San Jose, CA, USA; Catalog No. 638315) according to the manufacturer’s instructions. The resulting cDNA was used for quantitative PCR (qRT-PCR) to quantify the expression of selected miRNAs, as well as *ITGB1*, *TIMP3*, and *BRAF* genes.

### 2.4. RNA Extraction from Fresh Tumor Samples

For fresh frozen ovarian cancer tissues, RNA, DNA, and protein were extracted from fourteen samples (0.03 g each) obtained from patients of two ethnic groups using the AllPrep^®^ DNA/RNA/Protein Mini Kit (QIAGEN; Catalog No. 80004) according to the manufacturer’s instructions, with minor modifications as previously described [21]. Tissue samples were lysed in Buffer RLT supplemented with β-mercaptoethanol (BME). The lysate was first passed through an AllPrep^®^ DNA spin column to isolate DNA; the flow-through containing RNA and protein was then applied to an RNA spin column for RNA purification. RNA was eluted in 50 μL of RNase-free water and quantified using a NanoDrop™ One Microvolume UV-Vis Spectrophotometer (Thermo Fisher Scientific, Waltham, MA, USA). DNA was eluted from the DNA column using 50 μL of heated Buffer EB and similarly quantified. Protein was precipitated from the remaining flow-through using Buffer APP, and the resulting pellet was stored at −80 °C for future studies but was not analyzed further in this work. The extracted RNA was reverse-transcribed to cDNA, which was used for qPCR quantification of selected miRNAs and the expression of *ITGB1*, *TIMP3*, and *BRAF*.

### 2.5. Quantitative Real-Time PCR (qRT-PCR)

Quantitative real-time PCR (qRT-PCR) was conducted to assess miRNA and gene expression using cDNA prepared from a total of 50 samples, including 19 FFPE serous ovarian carcinoma (SOC) samples and 31 fresh tumor tissues. The analysis was optimized according to previously established protocols [21]. Expression levels of miR-192-5p, miR-30d, hsa-miR-16-1-5p, miR-143-3p, and miR-20a-5p were quantified using primer sets specific to each miRNA, with U6 snRNA (Clontech, Mountain View, CA, USA) serving as the internal control (Table 1). PCR reactions were carried out in quadruplicate using SYBR Green Master Mix (Bio-Rad, Hercules, CA, USA) on a CFX96 Touch Real-Time PCR Detection System (Bio-Rad Laboratories, Oslo, Norway). Additionally, validation of *TIMP3*, *BRAF*, and *ITGB1* mRNA expression, along with their associated miRNAs (miR-192-5p, miR-30d-5p, miR-143-3p), was performed using RNA isolated with the miRNeasy Mini Kit (QIAGEN). Reverse transcription was carried out using the High-Capacity cDNA Reverse Transcription Kit (Applied Biosystems Inc., 5791 Van Allen Way, Carlsbad, CA, USA, Catalog #43-688-14), and qRT-PCR was performed with SYBR Green Master Mix (Applied Biosystems) on a QuantStudio™ 6 Flex Real-Time PCR System. Gene expression was normalized to GAPDH, while miRNA expression was normalized to U6 snRNA, in accordance with the MIQE (Minimum Information for Publication of Quantitative Real-Time PCR Experiments) guidelines. Primer specificity and efficiency (90–110%) were verified by standard curve analysis using serial dilutions of pooled cDNA, and melting curve analysis confirmed the absence of nonspecific amplification or primer-dimer formation. All reactions were run in triplicate for both biological and technical replicates. Samples with a Ct standard deviation greater than 0.3 were excluded. Relative expression levels were calculated using the 2^−ΔΔCt^ (Livak) method [22], ensuring consistent normalization across all tissue types. Data were analyzed using SAS version 9.4 (SAS Institute Inc., Cary, NC, USA).

### 2.6. Statistical Analysis

To determine whether the observed expression differences in prognostic genes (*TIMP3*, *BRAF*, *ITGB1*) and associated microRNAs (miR-192-5p, miR-30d-5p, miR-143-3p) between African American and Caucasian patients were independent of clinical characteristics, we performed multivariate regression analyses.

Statistical Model: Multiple linear regression was used to assess differential expression by race, while adjusting for age (pre- and postmenopausal) at diagnosis, and tumor stage (FIGO classification). Survival Analysis: Multivariate Cox proportional hazards models were applied to evaluate the prognostic value of each marker after adjusting for the same covariates. Model Validation: Proportional hazards assumptions were verified using Schoenfeld residuals. Covariate inclusion was determined through backward selection with *p* < 0.1 as the entry criterion. Statistical significance was set at *p* < 0.05. All analyses were performed in R (version 4.3.1) using the survival and stats packages.

Given the demographic diversity between cohorts, we performed multivariate analyses to adjust for potential clinical confounders. Within the TCGA dataset, multiple linear regression was employed to assess differential gene and miRNA expression by race, while adjusting for age, tumor stage, and treatment type. In parallel, Cox proportional hazards models were used to assess the independent prognostic significance of key markers (*TIMP3*, *BRAF*, *ITGB1*, and associated miRNAs). This combined approach, conducting the racially diverse LLU cohort for cross-validation and the TCGA cohort for population-scale adjustment, ensured that observed molecular differences represent actual biological effects rather than sampling bias. The primary analysis of miRNA (miR-192-5p, miR-30D, hsa-miR-16-1-5p, miR-143-3p, miR-20a-5p) gene differential expression and correlated survival analysis in Caucasian versus African American ovarian cancer samples in TCGA was performed using the UALCAN online database platform [23]. Predicted prognostic markers associated with the selected miRNAs were identified using the Human Protein Atlas (www.proteinatlas.org) embedded in the UALCAN database. The association between predicted prognostic markers and target miRNAs was performed using miRTargetLink 2.0 [24] and Enrichr (https://maayanlab.cloud/Enrichr/) accessed on 7 February 2025) [25] platforms to establish interactive miRNA target gene and target pathway networks.

Changes in Cycle Threshold (ΔCt) values obtained from qRT-PCR were analyzed for relative expression using Microsoft Excel. Further statistical analyses were conducted using SAS 9.4 (SAS Institute Inc., Cary, NC, USA) and R (version 4.42). We performed linear regression analyses to assess the associations between the relative expression levels of miR-192-5p, miR-30D, hsa-miR-16-1-5p, miR-143-3p, miR-20a-5p, and key independent variables, including Ethnicity, Stage at Diagnosis, Age at Diagnosis, Overall Survival (OS_ Months), and OS_ Status (Supplementary Table S1). The normality of continuous quantitative variables was tested using the Shapiro–Wilk test, and statistical significance was defined as a *p*-value of less than 0.05. A Wilcoxon test was used to assess survival differences between ethnic groups. The association between two categorical variables was evaluated using Fisher’s exact test.

## 3. Results

### 3.1. Differential Expression of Predicted Prognostic Markers in Ovarian Cancer Tissues from the TCGA Cohort

**Figure 1:**
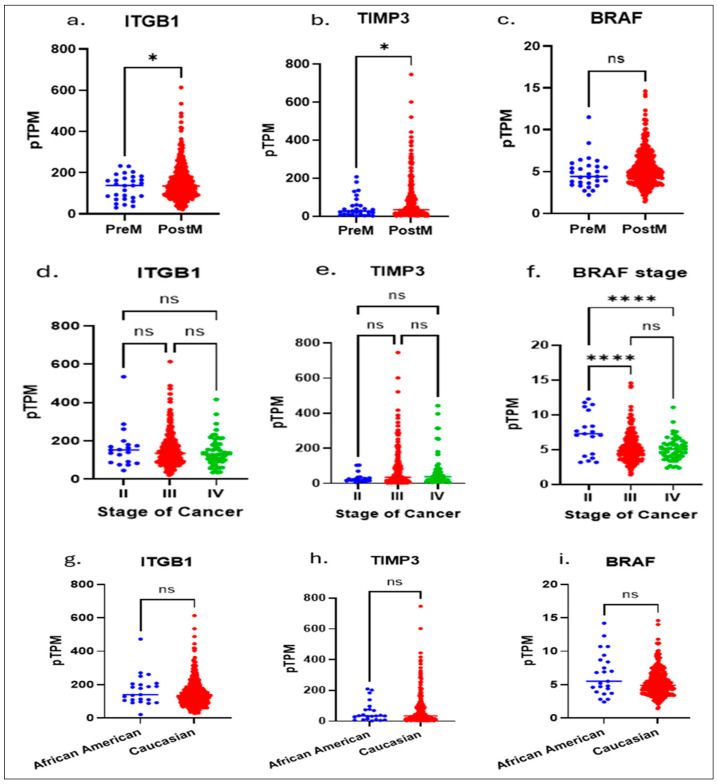
The Cancer Genome Atlas (TCGA) and The Human Protein Atlas, which provide comprehensive genomic and proteomic profiles across multiple cancer types, we performed an integrative bioinformatic analysis. Multivariate analysis of the TCGA data showed: significant differences between pre and postmenopausal expressions of *ITGB1* (**a**) and TIPMP3 (**b**) genes, whereas no significance in *BRAF* (**c**); in stage II to Stage IV: there are no significant differences shown in expressions of *ITGB1* (**d**) and TIPMP3 (**e**) genes; a higher significance in *BRAF* expression (**f**) between Stage II to Stage III and Stage II to Stage IV is shown. No racial significance was observed in *ITGB1* (**g**), *TIMP3* (**h**), and *BRAF* (**i**) expressions. An unpaired *t*-test with Welch’s correction (**a**–**c**,**g**–**i**) or One-way Anova (**d**–**f**) is performed. * Indicates a *p* ≤ 0.02 and **** indicates *p* < 0.0001.; ns, not significant, indicates a *p* > 0.05 or more.

To investigate race-associated molecular differences in serous ovarian cancer (SOC), we aimed to identify key genetic markers that predict overall survival. Using publicly available datasets from The Cancer Genome Atlas (TCGA) and The Human Protein Atlas, which provide comprehensive genomic and proteomic profiles across multiple cancer types, we performed an integrative bioinformatic analysis. This analysis identified *ITGB1*, *TIMP3*, and *BRAF* as critical prognostic genes whose expression patterns were significantly associated with patient survival (Figure 1a–i). The target genes analyzed in this study (e.g., *ITGB1*, *TIMP3*, and *BRAF*) were selected based on their strong prognostic relevance identified from cBioPortal survival analyses of high-grade serous ovarian carcinoma. We performed a multivariate analysis of *ITGB1*, *BRAF*, and *TIMP3* using the TCGA database and correlated the results with age, staging, and race. Notably, these findings revealed distinct molecular signatures between pre- and postmenopausal OSC patients (*ITGB1* and *TIMP3*) and staging (*BRAF*).

Although there were notable differences in *TIMP3* and *ITGB1* between African American and Caucasian patients, with no change in *BRAF* expression, this emphasizes the need to understand how such ancestry-related genetic variations may contribute to differential therapeutic responses and inform the development of precision, ancestry-informed treatment strategies.

To further explore the post-transcriptional regulatory mechanisms underlying these race-associated disparities, we examined the relationship between key microRNAs (miRNAs) and the previously identified prognostic genes (*TIMP3*, *BRAF*, and *ITGB1*) using integrated analyses of the TCGA and Human Protein Atlas datasets. This approach allowed us to identify potential miRNA–mRNA regulatory interactions that may modulate gene expression and influence survival outcomes across racial groups.

### 3.2. Identification of Regulatory microRNAs (miRNAs) Associated with Prognostic Genes and Race-Dependent Expression Patterns in HGSOC

To elucidate the post-transcriptional mechanisms contributing to the differential expression of prognostic genes identified in Section 3.1, we next investigated microRNAs (miRNAs) that may regulate *TIMP3*, *BRAF*, and *ITGB1* in high grade serous ovarian cancer (HGSOC). This analysis aimed to identify regulatory miRNA–mRNA pairs potentially underlying ancestry-associated disparities in gene expression and survival. Using integrated analyses of TCGA and Human Protein Atlas datasets, we characterized specific miRNAs that showed significant inverse correlations with these prognostic genes, providing insight into race-dependent molecular networks that may influence HGSOC prognosis and treatment response.

**Figure 2:**
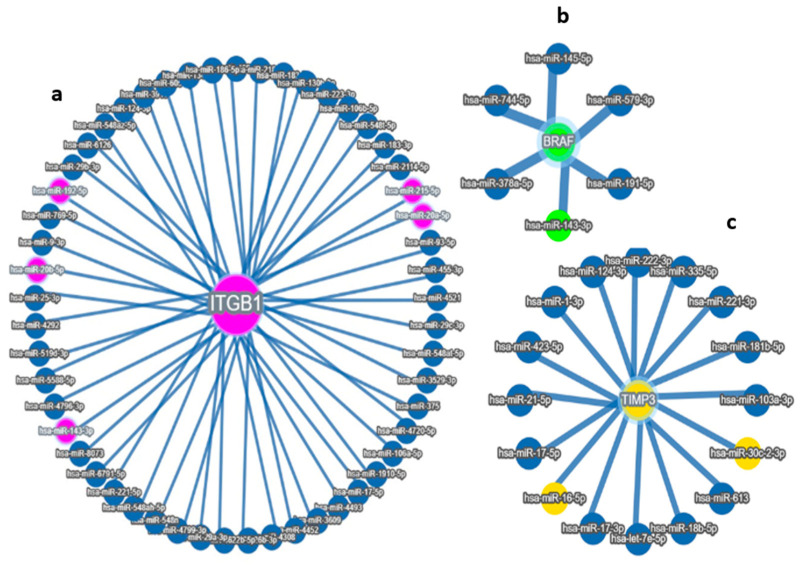

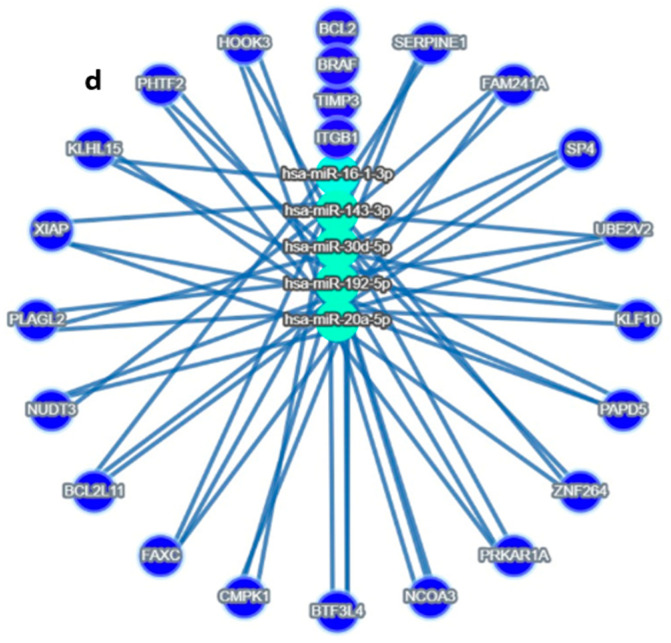
Target MicroRNas Linked to Prognostic Markers: (**a**–**d**): (**a**) miR-192, hsa-miR-16-5p, miR-143-3p, and miR-20a-5p are linked to *ITGB1*, (**b**) miR-143-3p/miR-145-5p is linked to *BRAF*, (**c**) hsa-miR-16-5p and miR30c/d are linked to *TIMP3* prognostic markers, (**d**) Integrated miRNA–gene interaction network associated with serous ovarian cancer prognosis. The diagram illustrates predicted and validated regulatory interactions between key microRNAs (miR-192-5p, miR-30d, miR-16-5p, miR-143-3p, miR-20a-5p) and their target prognostic genes (*ITGB1*, *TIMP3*, *BRAF*). Circles represent miRNAs; ovals represent target genes. Solid lines denote experimentally validated interactions (from miRTargetLink 2.0); Color coding: Turquoise Blue = miRNA nodes; Royal blue = prognostic genes; edge thickness corresponds to interaction with confidence. The figure highlights key regulatory axes-miR-192 → *ITGB1*, miR-30d → *TIMP3*, and miR-143 → *BRAF*-that may influence racial disparities and survival outcomes in serous ovarian cancer.

Using integrated target prediction tools (e.g., TargetScan, miRDB, and miRTarBase), we identified several miRNAs with high-confidence binding sites in the 3′ untranslated regions (3′UTRs) of these genes. Among these, five miRNAs—miR-192-5p, miR-30D, hsa-miR-16-1-5p, miR-143-3p, and miR-20a-5p—emerged as strong candidates based on both bioinformatic prediction and prior literature linking them to tumor progression and survival in ovarian and other cancers. Through integrative bioinformatic screening and correlation analyses, we identified three key miRNAs—miR-192-5p, miR-30d-5p, and miR-143-3p—as potential post-transcriptional regulators of the prognostic genes *ITGB1*, *TIMP3*, and *BRAF*, respectively. Expression profiling across the TCGA ovarian cancer cohort demonstrated that these miRNAs exhibited significant inverse correlations with their predicted target genes, supporting their potential regulatory interactions. Specifically, miR-192-5p showed a strong negative association with *ITGB1*, miR-30d-5p with *TIMP3*, and miR-143-3p with *BRAF* expression levels. Differential expression analysis further revealed that these miRNA–mRNA pairs displayed distinct patterns between African American and Caucasian patients, suggesting ancestry-related regulatory divergence. Collectively, these findings implicate miR-192-5p, miR-30d-5p, and miR-143-3p as critical modulators of gene expression networks influencing ovarian cancer progression, highlighting their potential as race-informed prognostic biomarkers and therapeutic targets in HGSOC. These miRNA–gene interactions suggest potential regulatory mechanisms that may influence tumor behavior and patient prognosis (Figure 2a–d). These results support a potential race-associated regulatory axis involving both miRNAs and their target prognostic genes. The observed expression patterns reinforce the biological relevance of these markers and provide further evidence of molecular differences that may underlie racial disparities in ovarian cancer progression and outcomes. These miRNA–mRNA interactions suggest a mechanistic framework through which differential miRNA expression may influence the activity of critical prognostic genes, ultimately impacting tumor behavior and patient outcomes.

### 3.3. Enrichment Pathway Analysis of miRNAs Linked to Prognostic Markers

**Figure 3:**
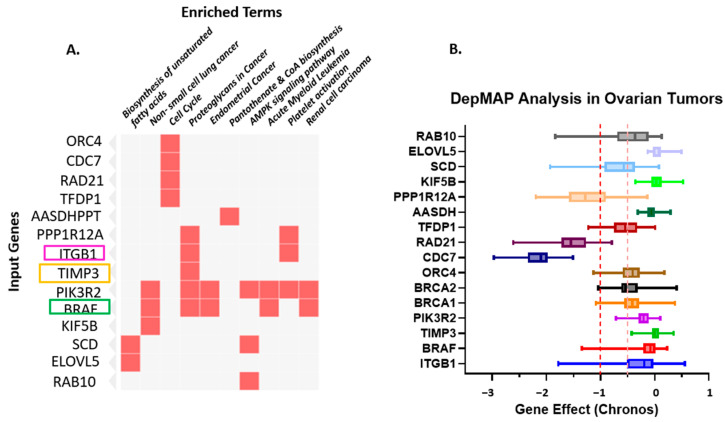
(**A**). Gene–Pathway Enrichment Heatmap. The heatmap illustrates the relationship between input genes and enriched biological pathways or functional terms. The rows represent the input genes (e.g., ORC4, CDC7, RAD21, TFDP1, PIK3R2, *BRAF*, KIF5B). At the same time, the columns correspond to enriched terms identified through pathway enrichment analysis (e.g., Biosynthesis of unsaturated fatty acids, non-small cell lung cancer, Cell cycle, Proteoglycans in cancer, Endometrial cancer, Pantothenate and CoA biosynthesis, AMPK signaling pathway, Acute myeloid leukemia, Platelet activation, Renal cell carcinoma). Red squares indicate a gene–pathway association, where a given gene contributes to the enrichment of that pathway. The intensity of the red color denotes the strength of the association or significance level (darker shades represent higher relevance or enrichment scores). This visualization highlights key genes (*TIMP3*, *BRAF*, and *ITGB1*, among the top 10 genes) that participate in multiple cancer-related and metabolic pathways, suggesting potential functional hubs and crosstalk among signaling networks. highlighting *ITGB1*, *TIMP3* and *BRAF*. (**B**). Depmap analysis of Ovarian Tumors: Dependency of Ovarian Tumors on the Prognostic Markers. The dependency on prognostic markers (*ITGB1*, *TIMP3*, and *BRAF*) compared to other important factors in Ovarian cancer was analyzed using CRISPRi/RNAi data available from the Cancer Dependency MAP website. A gene effect value closer to “0” indicates no survival dependency, whereas “−1” indicates very high dependency. Different colors are used to identify different genes. The red dotted line indicates the cut-off value of downregulated genes; orange dotted lines indicate overlapping expression of all genes.

To gain a deeper insight into the functional relevance of the identified miRNAs—miR-192-5p, miR-30D, hsa-miR-16-1-5p, miR-143-3p, and miR-20a-5p—and their role in regulating key prognostic genes (*TIMP3*, *BRAF*, and *ITGB1*), we performed pathway enrichment analysis. The goal was to elucidate the biological processes and signaling pathways that these miRNAs may influence in the context of SOC. Using the Enrichr online tool, which integrates multiple pathway databases including KEGG, Reactome, and BioCarta, we analyzed the top predicted gene targets for each miRNA. A total of 81 unique gene targets were compiled across the five miRNAs for enrichment analysis. The analysis revealed several significantly enriched pathways (adjusted *p* < 0.05). Using the Enrichr online platform, we performed gene enrichment analysis on the 81 genes identified from the miRNA enrichment analysis. This allowed us to generate a clustergram highlighting the most significant functional associations. Specifically, the clustergram (Figure 3) and summary table (Table 2) present the top 10 most significantly enriched genes and pathways, highlighting the strong involvement of these miRNAs in cancer-relevant biological networks.

These results provide insight into the biological processes and signaling pathways that may be regulated by the identified miRNAs, supporting their relevance in disease mechanisms and prognostic significance. This visualization highlights key genes (*TIMP3*, *BRAF*, and *ITGB1*, among the top 10 genes) that participate in multiple cancer-related and metabolic pathways, suggesting potential functional hubs and crosstalk among signaling networks. PIK3R2, the regulatory subunit p85β of the PI3K complex, is increasingly recognized as a key mediator of PI3K/AKT signaling in ovarian cancer. Its overexpression enhances downstream AKT phosphorylation, thereby promoting cell survival and chemoresistance. In Supplemental Figure S1, we present PIK3R2 expression and survival analyses derived from TCGA ovarian cancer datasets using the cBioPortal and GEPIA platforms. Although the association did not reach statistical significance, the data revealed a discernible trend toward racial variation in PIK3R2 expression, with comparatively higher levels observed in African American patients than in Caucasian counterparts. Despite the absence of a significant correlation with overall survival (*p* = 0.16), these findings suggest potential ancestry-associated differences in PIK3R2 expression that may contribute to biological heterogeneity in ovarian carcinoma. Further studies with larger and racially diverse cohorts are warranted to validate these observations and clarify the role of PIK3R2 in ovarian cancer disparities.

Based on these public databases, we have included the Loma Linda University (LLU) cohort and followed with the TCGA ovarian cancer cohorts, which highlight the need to validate molecular findings across independent and demographically distinct datasets. The LLU cohort, although smaller in size, comprises a racially diverse group of patients with well-characterized clinical and pathological data. In contrast, the TCGA dataset offers a much larger sample size (*n* = 444) and serves as a robust reference for population-level expression trends. After multivariate adjustment for clinical covariates in the TCGA dataset, *TIMP3*, *BRAF*, and *ITGB1* remained significantly associated with survival and demonstrated consistent expression differences between African American and Caucasian patients (adjusted *p* < 0.05). Similarly, miR-192-5p, miR-30d-5p, and miR-143-3p preserved strong inverse correlations with their target genes, confirming that these ancestry-related molecular patterns are biologically driven and reproducible across independent cohorts (LLU and TCGA).

### 3.4. Qualitative and Quantitative Clinical and Pathological Characterization of the Patients in the LLU Cohort

All patients included in the LLU were histologically confirmed to have high-grade serous ovarian cancer, as detailed in Supplementary Table S1. Among the 35 patients, eight were diagnosed at stage I, four at stage II, 19 at stage III, and four at stage IV, based on the FIGO staging criteria. All patients underwent primary debulking surgery, achieving optimal cytoreduction, and subsequently received standard first-line chemotherapy consisting of carboplatin and paclitaxel. Although the average overall survival rate was 47.2 years in African American patients versus 61.2 years in Caucasian patients, no statistically significant difference was observed in overall survival between the two groups, as demonstrated by the Kaplan–Meier survival analysis shown in Supplementary Figure S2. The log-rank test returned to a *p*-value of 0.75, indicating no difference in survival between the two ethnic groups. This suggests that, within this cohort and treatment context, race did not significantly impact overall survival outcomes. This lack of significance is likely due to the limited sample size in the LLU cohort, which may have reduced the statistical power to detect meaningful differences. Future studies with larger and more balanced cohorts may help clarify whether any true disparities in survival outcomes exist across ethnic groups.

**Figure 4:**
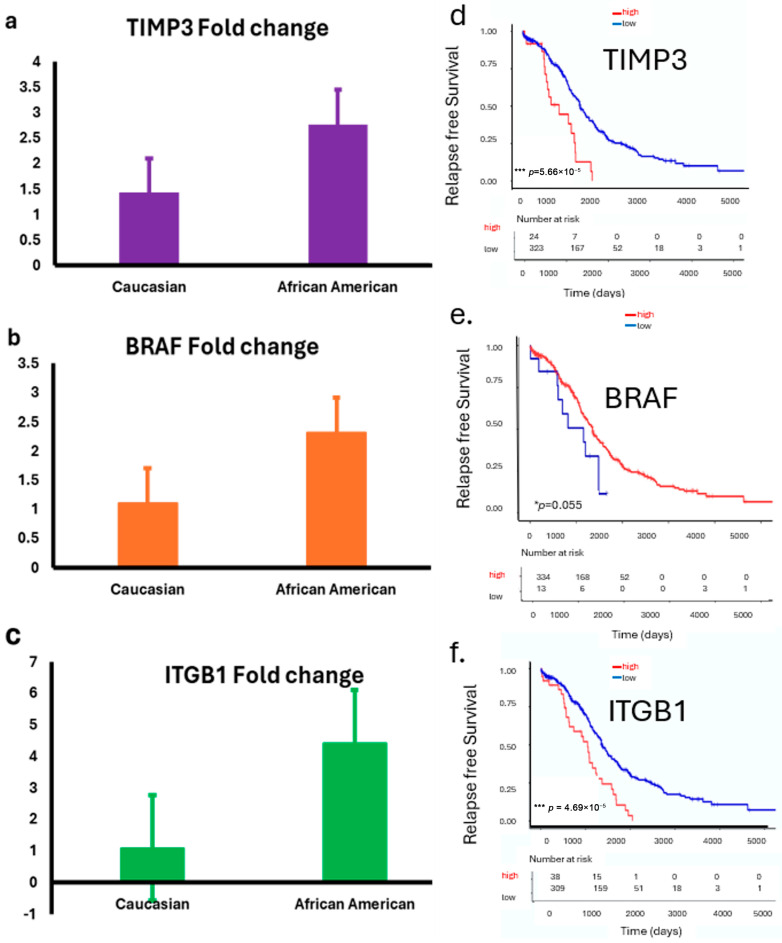
(**a**–**f**): Expression of *TIMP3*, *BRAF*, and *ITGB1* in LLU and TCGA ovarian cancer cohorts stratified by race. (**a–c**): Bar plots show relative expression (fold changes) of *TIMP3* (*p* = 0.02) (**a**); *BRAF* (*p* = 0.84, not significant) (**b**), and *ITGB1* (*p* = 0.01) (**c**) (two-tailed *t*-test) in ovarian tumor samples from the LLU cohort, comparing Caucasian and African American patients. Data are presented as mean ± SEM. (**d**–**f**): TCGA data analysis revealed a significant correlation between *ITGB1*, *TIMP3*, and *BRAF* gene expression and survival. These prognostic gene expression patterns (high or low) are significantly associated with patient survival: higher expression of *TIMP3* (*** *p* = 0.000046) (**d**), lower *BRAF* (* *p* = 0.05) (**e**), and higher *ITGB1* (*** *p* = 0.000056) (**f**) correlates with poor survival. * *p* < 0.05; *** *p* < 0.001. The red line graph indicates high expression, and the blue line indicates low expression.

We examined the expressions of *TIMP3*, *BRAF*, and *ITGB1* in ovarian tumor samples from both the LLU cohort and, subsequently, confirmed these findings using the TCGA database, stratified by race. In the LLU cohort, relative expression analysis by quantitative PCR (qPCR) revealed differential patterns between Caucasian and African American patients. TIMP3 (* p = 0.02), *BRAF* (*p* = 0.84), and *ITGB1* (* *p* = 0.01) expression was relatively higher in African American tumors compared with Caucasian tumors (Figure 4a–f). To validate these findings, we analyzed RNA-seq–based transcript levels in the TCGA ovarian cancer cohort. *TIMP3* expression was consistently lower in African American patients compared with Caucasian patients (Figure 4d). In contrast, *BRAF* and *ITGB1* expressions are elevated across the three racial groups (Figure 4e,f). These findings provide compelling evidence that *TIMP3*, *BRAF*, and *ITGB1* may not only serve as prognostic biomarkers in HGSOC but also play a role in influencing ovarian cancer outcomes.

### 3.5. Survival Analysis Using Kaplan–Meier Survival Curves

Survival analysis of the TCGA ovarian cancer cohort revealed that higher expression levels of *TIMP3*, *BRAF*, and *ITGB1* were significantly associated with poorer overall survival (Figure 4d,f). Patients with elevated *TIMP3* expression had a reduced survival rate compared with those with lower expression (** *p* = 0.000046). In contrast, low BRAF expression correlated with worse outcomes (* *p* = 0.05). Additionally, increased *ITGB1* expression was strongly associated with the poorest survival (*** *p* = 0.000056). These findings suggest that elevated expressions of *TIMP3* and *ITGB1* and downregulated *BRAF* may serve as unfavorable prognostic markers in ovarian cancer.

### 3.6. Differential Expression of miR-192, miR-143-3p, miR-30D, hsa-miR-16-5p and miR-20a-5p in LLU by qRT-PCR and TCGA Ovarian Cancer Cohorts

**Figure 5:**
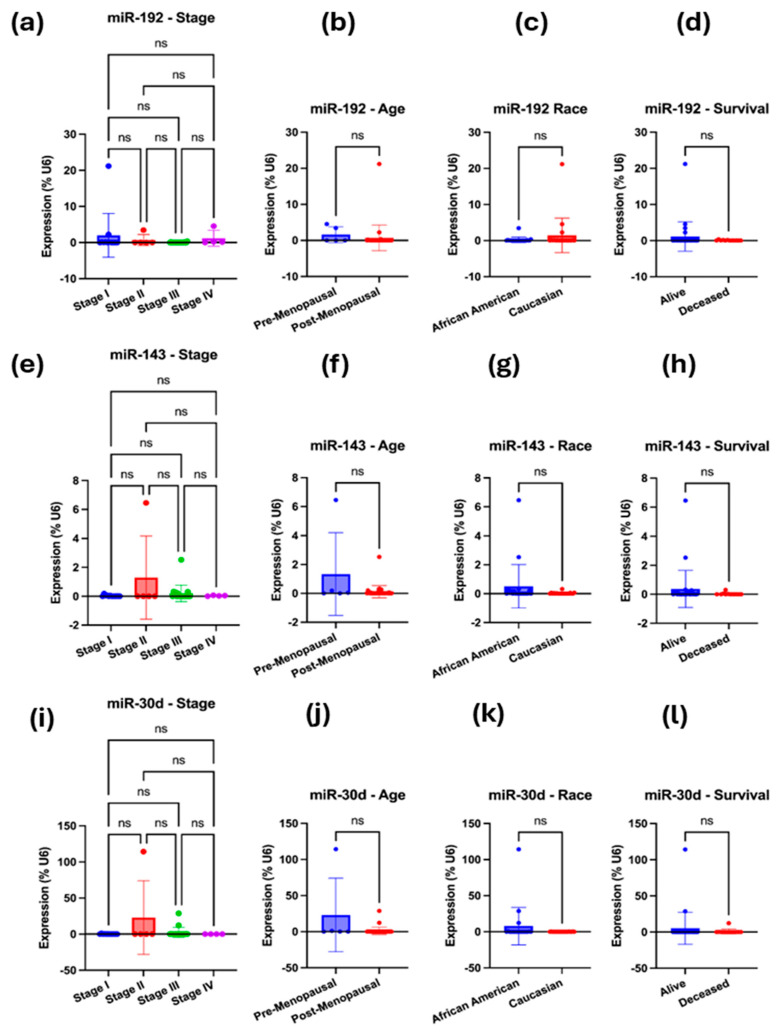
(**a**–**l**): qRT-PCR-based gene expression analysis of miR-192, miR-143-5p, and miR-30D. (**a**–**d**) Multivariate analysis was performed for miR-192-5p, i.e., expression vs. cancer stage (**a**), menopausal status (**b**), race (**c**), or survival outcome (**d**). (**e**–**h**) Multivariate analysis was performed for miR-143-5p, specifically examining its expression in relation to cancer stage (**e**), menopausal status (**f**), race (**g**), or survival outcome (**h**). (**i**–**l**) Multivariate analysis was performed for miR-30D, examining its expression in relation to cancer stage (**i**), menopausal status (**j**), race (**k**), and survival outcome (**l**). In each case, the expression was normalized to the U6 reference. A one-way ANOVA or an unpaired *t*-test with Welch’s correction was performed. Not significant (ns), when the *p*-value is higher than 0.05.

**Figure 6:**
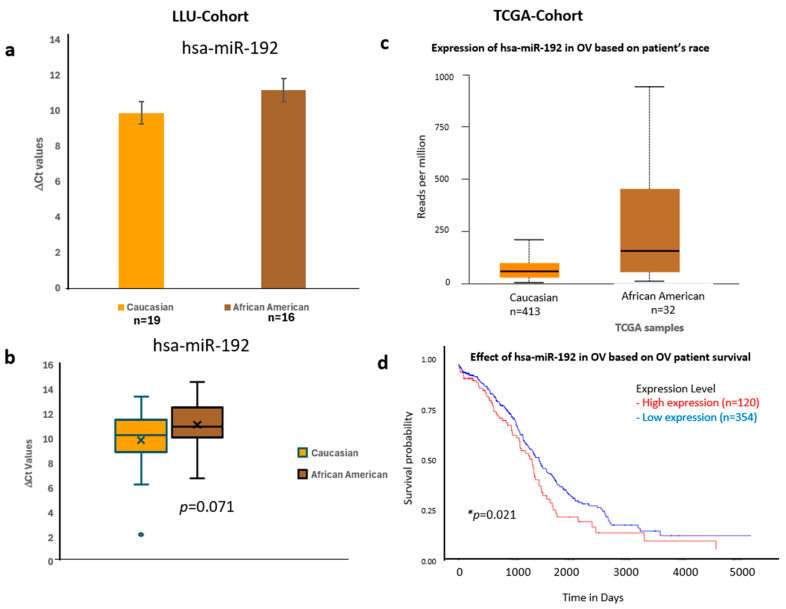
Comparative expression and survival analysis of miR-192 in ovarian carcinoma across LLU and TCGA cohorts. (**a**–**d**) miR-192: Panels (**a**,**b**) show miR-192 expression in the LLU cohort, comparing Caucasian (*n* = 19) and African American (*n* = 16) patients, revealing higher expression in African Americans (ns, *p* = 0.071). Panel (**c**) presents TCGA data showing a similar trend toward elevated expression in African American ovarian cancer samples. Panel (**d**) displays the TCGA survival curve, indicating that higher miR-192 expression is associated with poorer overall survival (* *p =* 0.021). ns, not significant; *, significant when *p* < 0.05. Error bars represent mean ± SEM. Statistical analyses were performed using Student’s *t*-test or log-rank test where appropriate.

**Figure 7:**
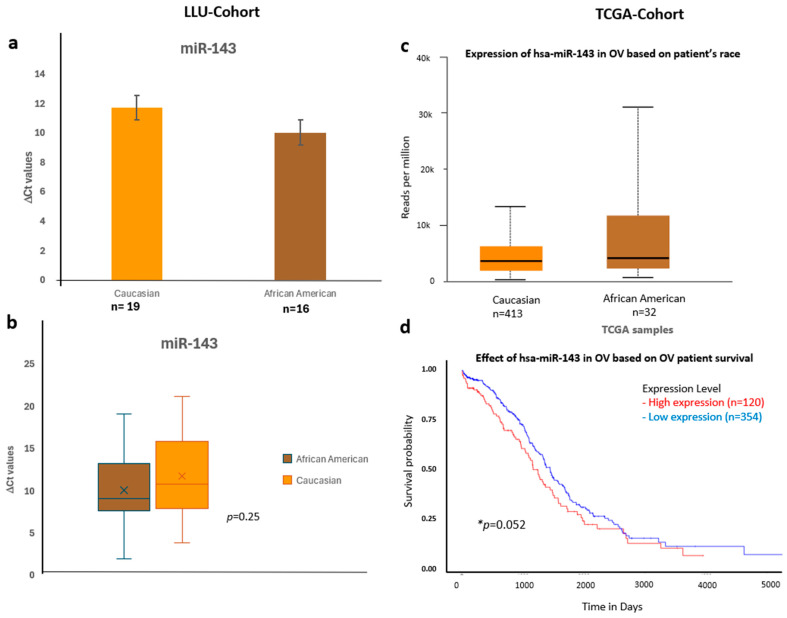
Comparative expression and survival analysis of miR-143 in ovarian carcinoma across LLU and TCGA cohorts. (**a**–**d**) miR-143: Panels (**a**,**b**) show LLU cohort expression data comparing Caucasian and African American patients (ns, *p* = 0.25), with TCGA validation in panel (**c**), which also demonstrates relatively higher miR-143 expression in African American samples. The TCGA survival analysis (**d**) indicates that elevated miR-143 expression is associated with decreased overall survival (* *p* = 0.05). *, significant when *p* < 0.05; ns, not significant; *, Error bars represent mean ± SEM. Statistical analyses were performed using Student’s *t*-test or log-rank test where appropriate.

**Figure 8:**
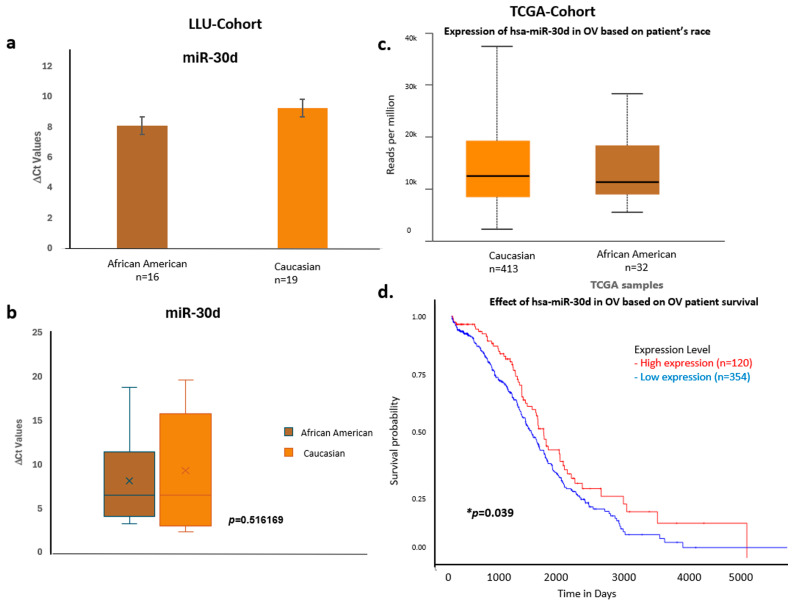
Comparative expression and survival analysis of miR-30d in ovarian carcinoma across LLU and TCGA cohorts. (**a**–**d**) miR-30d: Panels (**a**,**b**) depict LLU cohort data showing similar miR-30d expression levels across racial groups (ns, *p* = 0.516), consistent with TCGA data (**c**), which also show no significant racial difference. The TCGA survival plot (**d**) shows a significant association between miR-30d expression and overall survival (* *p* = 0.039). *, denotes significance when *p* < 0.05. Error bars represent mean ± SEM. Statistical analyses were performed using Student’s *t*-test or log-rank test where appropriate.

To validate the bioinformatically predicted microRNAs (miR-192-5p, miR-143-3p, miR-30d-5p, hsa-miR-16-5p, and miR-20a-5p) regulating *ITGB1*, *TIMP3*, and *BRAF*, we compared their expression profiles between the LLU-OV and the TCGA-OV datasets. Expression levels were normalized to internal controls and analyzed using qRT-PCR for LLU samples and RNA-seq for TCGA data. The five miRNAs—miR-192, miR-30D, hsa-miR-16-5p, miR-143-3p, and miR-20a-5p—were selected based on their predicted targeting of key prognostic genes (*TIMP3*, *BRAF*, and *ITGB1*); enrichment pathway analysis implicating them in cancer-relevant signaling pathways. To determine whether the expression of selected microRNAs—miR-192-5p, miR-30D, hsa-miR-16-1-5p, miR-143-3p, and miR-20a-5p—differed between the two ethnic groups represented in the LLU cohort, we conducted qRT-PCR (Figure 5, Figure 6, Figure 7 and Figure 8). Multivariate analysis of their expressions vs. cancer stage (a), menopausal status (b), race (c), or survival outcome (d) was determined from qRT-PCR-based gene expression analysis of miR-192, miR-143-5p, and miR-30D (Figure 5a–l). Our analysis revealed trends of differential expression patterns of these miRNAs between African American and Caucasian patients (no significance).

To validate these findings, we confirmed the qPCR data by comparing it with expression trends observed in a larger cohort from TCGA (Figure 6, Figure 7 and Figure 8) (*n* = 444). miR-192, miR-143, miR-30D, hsa-miR-16-5p, and miR-20a-5p showed higher expression in African Americans compared to Caucasians, with a significant correlation with poor survival (*p* < 0.05). The consistency in expression trends across both cohorts reinforces the robustness of our results. These data are presented in Figure 6a–d, Figure 7a–d and Figure 8a–d, and Supplementary Figures S3 and S4. Collectively, these results highlight race-associated trends in miRNA expressions, particularly miR-192 and miR-143, that may contribute to differential ovarian cancer outcomes, warranting further validation in larger, racially diverse cohorts.

## 4. Discussion

The identification of reliable biomarkers for disease progression remains essential for improving survival outcomes in ovarian cancer (OC) patients [26]. In this study, we identified novel prognostic microRNA–gene regulatory networks associated with overall survival in serous ovarian cancer (SOC). Through integrative bioinformatic analysis of The Cancer Genome Atlas (TCGA) dataset and validation using the Loma Linda University (LLU) patient cohort, we demonstrated that *ITGB1*, *TIMP3*, and *BRAF* function as key molecular determinants of SOC prognosis. Among these, elevated *ITGB1* expression showed the strongest association with reduced survival, followed by *TIMP3*, whereas higher *BRAF* expression correlated with improved outcomes. Predicted upstream microRNAs targeting these genes included miR-192-5p, miR-30d-5p, miR-16-5p, miR-143-3p, and miR-20a-5p. Of these, miR-192-5p emerged as the most clinically relevant, demonstrating a strong negative correlation with overall survival in TCGA data and marked upregulation in premenopausal patients compared with postmenopausal patients in the LLU cohort. By performing this, we validated the reproducibility and consistency of miRNA expression trends across datasets, confirmed whether race-associated expression differences observed in the LLU cohort, established broader relevance and generalizability of the identified miRNA biomarkers in SOC. This cross-cohort analysis provides a crucial validation step, enhancing the credibility of our findings and supporting the translational potential of these miRNAs as biomarkers of prognostic and racial significance.

The gene–pathway enrichment heatmap and DepMap analyses collectively emphasize *ITGB1*, *TIMP3*, and *BRAF* as central molecular hubs in ovarian cancer biology. These genes participate in multiple cancer-related and metabolic pathways—including cell cycle regulation, proteoglycan signaling, AMPK signaling, and biosynthesis of unsaturated fatty acids—indicating critical roles in tumor progression and intercellular communication networks. The DepMap analysis further supports their biological significance, showing that ovarian tumor cells display substantial dependency on these genes for survival and proliferation. Gene effect values approaching –1 suggest that suppression of *ITGB1*, *TIMP3*, or *BRAF* severely compromises tumor cell viability. Collectively, these data highlight these genes as potential therapeutic vulnerabilities and promising targets for pathway-directed or combinatorial treatment strategies designed to disrupt tumor survival mechanisms.

The genes identified in this study are intricately involved in extracellular matrix (ECM) remodeling, adhesion signaling, and oncogenic kinase activation—processes critical for invasion, metastasis, and chemoresistance. *ITGB1* (Integrin β1), a key component of focal adhesion complexes, mediates cell–matrix interactions and mechanotransduction. Aberrant *ITGB1* signaling enhances activation of the FAK, PI3K/AKT, and MAPK pathways, promoting tumor proliferation and survival [26,27]. Reduced miR-192-5p-mediated repression of *ITGB1* may amplify these signaling cascades, leading to increased ECM stiffness and aggressive tumor phenotypes.

*TIMP3*, a potent endogenous inhibitor of matrix metalloproteinases (MMPs) and ADAM proteases, preserves ECM structure and limits angiogenesis [28]. Downregulation of *TIMP3* has been linked to increased invasion and metastasis across multiple cancers [29]. Our data suggests that miR-30d-driven suppression of *TIMP3* may diminish ECM integrity and accelerate tumor dissemination. Interestingly, miR-30d has also been reported to act as a tumor suppressor through PI3K/AKT attenuation [30], indicating potential context-specific functions across SOC molecular subtypes. The regulatory subunit p85β of the PI3K complex, called PIK3R2, is increasingly recognized as a key mediator of PI3K/AKT signaling in ovarian cancer. Its overexpression enhances downstream AKT phosphorylation, thereby promoting cell survival and chemoresistance. Analysis of TCGA data revealed that while elevated PIK3R2 expression was not significantly associated with overall survival, African American patients exhibited higher expression levels compared to Caucasian patients. This trend, though not statistically significant, suggests that inclusion of a larger cohort of African American samples may be necessary to achieve statistical power and validate the observed disparity.

*BRAF*, a serine/threonine kinase and central effector of the MAPK/ERK pathway, though rarely mutated in serous ovarian carcinoma (SOC), may contribute to tumor progression through non-mutational overexpression [31]. Its regulation by miR-143-3p and miR-145-5p, as observed here, suggests possible post-transcriptional control of MAPK signaling within the ovarian tumor microenvironment. In TCGA data, higher *BRAF* expression correlated with improved survival. Concurrently, elevated miR-192-5p—previously associated with immune modulation, angiogenesis, and epithelial–mesenchymal transition (EMT)—may promote tumor aggressiveness by repressing adhesion inhibitors and activating pro-migratory pathways. These findings align with emerging evidence that tumor microenvironmental heterogeneity and immune–stromal dynamics critically influence ovarian tumorigenesis beyond genomic alterations [32,33].

Advances in proteomic technologies, particularly mass spectrometry (MS)-based proteomics and high-throughput protein arrays, have deepened our understanding of molecular signaling networks underlying OC progression. These approaches enable comprehensive profiling of protein expression, post-translational modifications, and dynamic signaling cascades that drive tumor heterogeneity and therapeutic resistance. Proteomic analyses of OC continue to reveal novel therapeutic targets and biomarkers, offering strategies to overcome drug resistance and improve patient outcomes. The strong concordance between computational predictions and experimental validation across both datasets in this study reinforces the biological validity of the identified regulatory interactions.

Gene ontology and pathway enrichment analyses (miRTargetLink2.0, Enrichr) revealed overlapping networks linking miR-192, miR-16-5p, miR-143-3p, and miR-20a-5p to *ITGB1*; miR-143-3p/miR-145-5p to *BRAF*; and miR-16-5p/miR-30c/d to *TIMP3*. We have added a focused subsection in the Discussion detailing how the miR-192 → *ITGB1* and miR-30d → *TIMP3* axes plausibly regulate extracellular-matrix (ECM) remodeling, adhesion/FAK–MAPK signaling, and protease control-pathways that were enriched in our Enrichr analysis (e.g., proteoglycans in cancer, cell cycle, MAPK signaling) and involve our three prognostic genes (*ITGB1*, *TIMP3*, *BRAF*) highlighted in the pathway heatmap.

These networks converged on ECM–receptor interaction, focal adhesion, and MAPK signaling pathways, all well-established mediators of tumor stiffness, invasion, and chemoresistance [34,35].

The identification of miR-192-5p as a prognostic biomarker holds important translational implications. Its overexpression in African American patients and association with poorer survival underscore its potential as an ancestry-informed prognostic marker and therapeutic target. Interventions such as miR-192 inhibition or blockade of downstream integrin/FAK–MAPK signaling could enhance treatment responsiveness in SOC. Likewise, modulating the miR-30d–*TIMP3* axis may help restore ECM homeostasis and suppress metastatic progression.

Incorporating miRNA–mRNA signatures into clinical workflows may significantly improve early detection, risk stratification, and personalized therapeutic decision-making. Functional validation using ovarian cancer cell lines, 3D organoid models, and patient-derived xenografts (PDXs) will be essential to confirm causality and therapeutic potential. Integration with spatial transcriptomics and proteomic profiling could further elucidate cell-type-specific regulation within the ovarian tumor microenvironment.

### Limitations and Future Directions

While this study integrates multi-cohort bioinformatic and experimental data, the relatively small LLU cohort limits statistical power. Larger, multi-institutional studies are necessary to validate ancestry-associated molecular differences and elucidate the mechanistic interplay between miRNAs and their target genes. Future efforts incorporating spatially resolved transcriptomics and single-cell multi-omics will yield deeper insights into the spatial and temporal dynamics of these regulatory networks. Currently, CA125 and HE4 remain the only FDA-approved biomarkers for epithelial ovarian cancer (EOC), yet their limited sensitivity-particularly in early-stage disease-highlights the need for improved diagnostic tools. Integrating the newly identified prognostic gene–miRNA markers with existing algorithms such as the Risk of Ovarian Malignancy Algorithm (ROMA) may enhance diagnostic precision and individualized risk assessment.

## 5. Conclusions

This study integrates experimental and bioinformatic analyses to uncover microRNA–gene regulatory networks contributing to racial disparities in serous ovarian cancer. Using TCGA and Loma Linda University cohorts, we identified three key prognostic genes—*ITGB1*, *TIMP3*, and *BRAF*—and their regulatory microRNAs miR-192-5p, miR-30d-5p, and miR-143-3p. These interactions (miR-192–*ITGB1*, miR-30d–*TIMP3*, miR-143–*BRAF*) are linked to poor overall survival and enriched in cancer-related pathways involving extracellular matrix remodeling, adhesion, and MAPK signaling. Notably, miR-192-5p expression strongly correlated with adverse outcomes and was elevated in African American patients. Collectively, this study defines a core regulatory axis—miR-192-5p/*ITGB1*/*TIMP3*/*BRAF*—that underpins differences in ovarian cancer progression and patient survival. Incorporating ancestry-specific molecular insights into ovarian cancer research represents a critical step toward future precision oncology and advancing health equity in women’s cancer care.

## Figures and Tables

**Table 1:** List of miRNAs and U6 control Primers.

miRNA	Forward Sequence	Reverse Sequence	Temperature (°C)
miR-30D	5′-TGT AAA CAT CCC CGA CTG GAA G-3′	5′-CTC AAC TGG TGT CGT GGA GTC GGC AAT TCA GTT GAG CTT CCA GTC-3′	65 °C
hsa-miR-16-1-5p	5′- ACA CTC CAG CTG GGT AGCAGCACG TAA ATA TTG GC-3′	5′-CTC AAC TGG TGT CGT GGA GTC GGC AAT TCA GTT GAG CGC CAA T-3′	70 °C
miR-192	5′- GCG CGC TGA CCT ATG AAT TG-3′	5′- AGT GCA GGG TCC GAG GTA TT-3′	60 °C
miR-143-3p	5′- TGA GAT GAA GCA CTG TAG CTC A-3′	5′-CTC AAC TGG TGT CGT GGA GTC GGC AAT TCAGTT GAG TCA ACA TCA G-3′	60 °C
miR-20a-5p	5′- GTA AAG TGC TTA TAG TGC AG-3′	5′-GTC GTA TCC AGT GCG TGT CG-3′	50 °C
U6 control	5′-GGA ACG ATA CAG AGA AGA TTA GC-3′	5′-TGG AAC GCT TCA CGA ATT TGC G-3′	60 °C
*BRAF*	5′-CATGAAGACCTCACAGTAAA-3′	5′-ACTGTTCAAACTGATGGGACCCAC−3′	68 °C
*ITGB1*	5′GGATTCTCCAGAAGGTGGTTTCG-3′	5′-TGCCACCAAGTTTCCCATCTCC-3′	60 °C
*TIMP3*	5′-TACCGAGGCTTCACCAAGATGC-3′	5′-CATCTTGCCATCATAGACGCGAC-3′	60 °C

**Table 2:** Top ten significant *p*-values and *q*-values for KEGG Human analysis.

Term	*p*-Value	*q*-Value	Overlap_Genes
Proteoglycans in cancer	0.001469	0.125586	[*ITGB1*, PPP1R12A, *TIMP3*, *BRAF*, PIK3R2]
Cell cycle	0.001620	0.125586	[ORC4, TFDP1, RAD21, CDC7]
Non-small cell lung cancer	0.003122	0.161288	[KIF5B, PIK3R2, *BRAF*]
Biosynthesis of unsaturated fatty acids	0.005325	0.206338	[ELOVL5, SCD]
Focal adhesion	0.009022	0.268648	[*ITGB1*, PPP1R12A, *BRAF*, PIK3R2]
Regulation of actin cytoskeleton	0.011887	0.268648	[*ITGB1*, PPP1R12A, *BRAF*, PIK3R2]
AMPK signaling pathway	0.012799	0.268648	[RAB10, SCD, PIK3R2]
Platelet activation	0.013972	0.268648	[*ITGB1*, PPP1R12A, PIK3R2]
Herpes simplex virus 1 infection	0.015599	0.268648	[ZNF320, ZNF460, ZNF85, PIK3R2, ZNF226, ZNF200]
Insulin signaling pathway	0.018196	0.275793	[PRKAR1A, *BRAF*, PIK3R2]

*p*-values, and *q*-values of significant terms in the selected library. The *q*-value is an adjusted *p*-value calculated using the Benjamini–Hochberg method for correction for multiple hypothesis testing.

## Data Availability

TCGA database (https://www.cancer.gov/ccg/research/genome-sequencing/tcga, accessed on 20 November 2024); UALCAN software (https://ualcan.path.uab.edu/, accessed on 20 November 2024); TargetScanHuman v8.0 (https://www.targetscan.org/vert_80/, accessed on 20 November 2024); Gene Ontology (https://ccb-compute.cs.uni-saarland.de/mirtargetlink2, accessed on 20 November 2024); http://amp.pharm.mssm.edu/Enrichr/, accessed on 20 November 2024. We will provide Whole slide images of H&E slides.

## Supplementary Materials

The following supporting information can be downloaded at https://www.mdpi.com/article/10.3390/diseases13110360/s1, Figure S1: (a–c). TCGA-cohort analysis of PIK3R2 expression and clinicopathological correlations in ovarian carcinoma; Figure S2: Kaplan–Meier survival analysis of ovarian cancer patients stratified by ethnicity in the LLU-cohort. Kaplan–Meier survival curves were generated using the LLU ovarian cancer (OV) cohort data to compare overall survival between African American and Caucasian patients. The analysis revealed no significant difference in survival between the two groups (p = 0.75). Although the average overall survival rate was 47.2 years for African American patients versus 61.2 years for Caucasian patients, no statistically significant difference was observed in overall survival between the two groups. Median survival times and overall survival probabilities were comparable, indicating that patient ethnicity did not significantly impact survival outcomes within this dataset; Figure S3: Comparative expression and survival analysis of miR-20a-5p in ovarian carcinoma across LLU and TCGA cohorts; Figure S4: Comparative expression and survival analysis of miR-16-1-5p in ovarian carcinoma across LLU and TCGA cohorts; Table S1: Clinical and demographic characteristics of the Loma Linda University (LLU) ovarian cancer patient cohort; Table S2: Lists of 81 prognostic genes analyzed by cBioportal.

## Abbreviations

The following abbreviations are used in this manuscript:

*ITGB1*	Integrin beta-1
*BRAF*	B-Raf Proto-Oncogene, Serine/Threonine Kinase
*TIMP3*	TIMP Metallopeptidase Inhibitor 3
LLU	Loma Linda University
