# Supplementary material for: MicroRNA Signatures in Serous Ovarian Cancer: A Comparison of Prognostic Marker Targets in African Americans and Caucasians
Source: Diseases. 2025 Nov 6;13(11):360. doi: 10.3390/diseases13110360 (PMC12650892; doi:10.3390/diseases13110360)
Supplement: Supplementary file 1 [file diseases-13-00360-s001.zip › diseases-3872579-supplementary.pdf]

Table S1. Clinical and demographic characteristics of the Loma Linda University (LLU) ovarian cancer patient cohort.

Patient Identity	Age at Diagnosis	Ethnicity	Stage at Diagnosis	Overall Survival Months	Overall Survival Status
**P001**	63.5	African American	III	23.2	Deceased
**P002**	76.8	African American	III	24	Alive
**P003**	57.5	Caucasian	III	37	Alive
**P005**	81.3	Caucasian	I	25.5	Deceased
**P006**	71	Caucasian	I	24	Alive
**P007**	79.6	Caucasian	III	2	Deceased
**P008**	62.9	Caucasian	IV	5	Deceased
**P009**	73.8	African American	IV	42	Deceased
**P010**	66.3	African American	II	24	Alive
**P011**	50.5	African American	I	63	Alive
**P012**	50.4	Caucasian	III	19	Alive
**P013**	63.2	Caucasian	I	27	Alive
**P014**	48.6	Caucasian	I	60	Alive
**P015**	39.5	Caucasian	IV	58	Alive
**P016**	68.1	Caucasian	III	54	Alive
**P017**	62.3	Caucasian	IV	58	Alive
**P018**	68.6	African American	III	36.5	Deceased
**P019**	57.1	African American	III	73	Alive
**P020**	51.3	African American	I	67	Deceased
**P024**	55.1	African American	III	26	Alive
**P025**	52	African American	III	21	Alive
**P028**	91	Caucasian	I	36	Deceased
**P029**	51	African American	I	116	Alive
**P030**	54	Caucasian	III	24	Deceased
**P032**	67	Caucasian	III	150	Alive
**P033**	44	African American	III	24	Alive
**P034**	74	Caucasian	II	96	Deceased
**P035**	49	Caucasian	III	12	Deceased
**P036**	61	African American	III	48	Alive
**P037**	37	Caucasian	III	147	Alive
**P038**	48	Caucasian	III	120	Deceased
**P039**	71	Caucasian	III	134	Alive
**P040**	63	African American	III	24	Alive
**P041**	86	Caucasian	II	136	Alive
**P042**	67	African American	II	97	Alive

	Average Age = **61.7 years**			Average OS = **55.2 Months**	

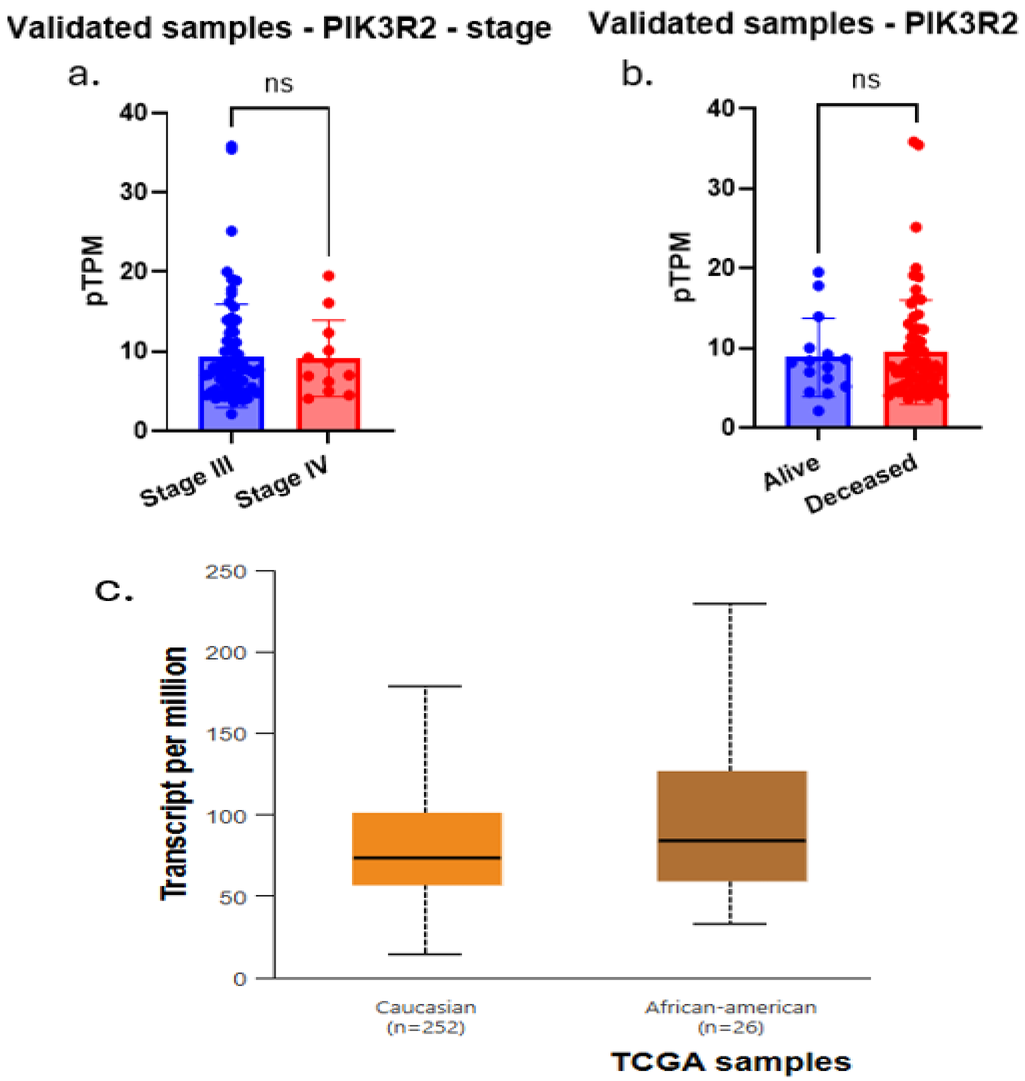

Figure S1. (a-c). TCGA-cohort analysis of PIK3R2 expression and clinicopathological correlations in ovarian carcinoma. (a) Comparison of PIK3R2 expression (pTPM) between Stage III and Stage IV ovarian cancer samples, showing no significant difference (ns). (b) Comparison of PIK3R2 expression (pTPM) between patients who were alive versus deceased, also showing no significant difference (ns). (c) Box plot representation of PIK3R2 transcript levels in TCGA ovarian cancer samples, comparing Caucasian (n = 252) and African American (n = 26) patients. Although African American patients exhibited a trend toward higher PIK3R2 expression, the difference was not statistically significant. Data were analyzed using TCGA-derived datasets and normalized as transcripts per million (TPM). Error bars represent mean ± SEM.

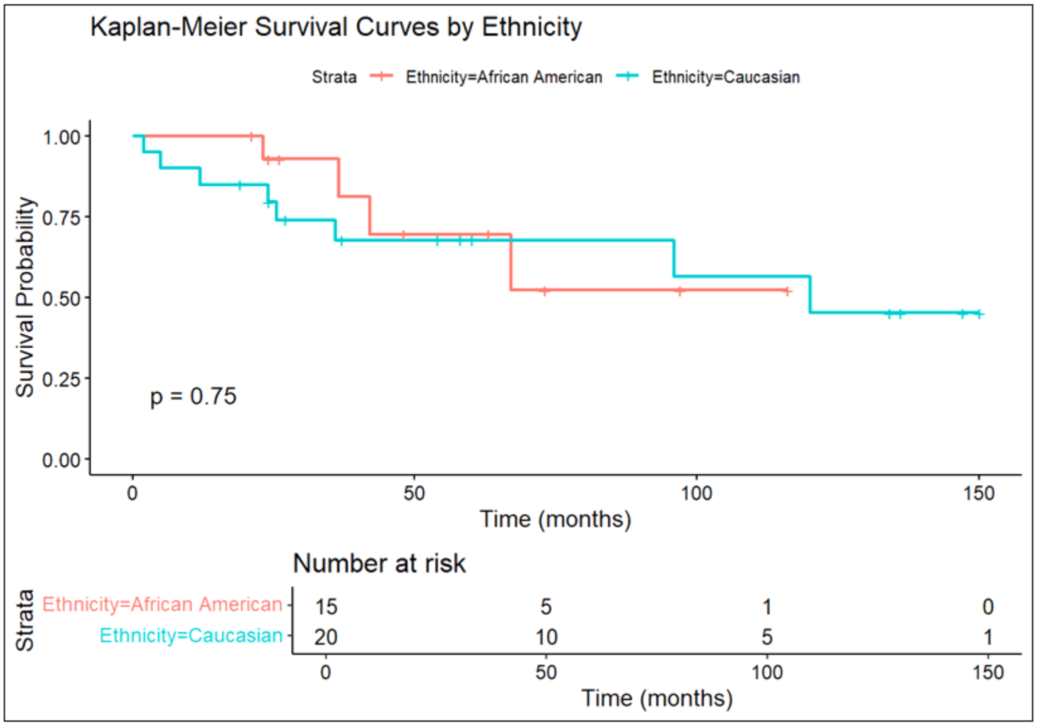

Figure S2. Kaplan–Meier survival analysis of ovarian cancer patients stratified by ethnicity in the LLU-cohort. Kaplan–Meier survival curves were generated using the LLU ovarian cancer (OV) cohort data to compare overall survival between African American and Caucasian patients. The analysis revealed no significant difference in survival between the two groups ($p = 0.75$). Although the average overall survival rate was 47.2 years for African American patients versus 61.2 years for Caucasian patients, no statistically significant difference was observed in overall survival between the two groups. Median survival times and overall survival probabilities were comparable, indicating that patient ethnicity did not significantly impact survival outcomes within this dataset.

Table S2: Lists of 81 prognostic genes analyzed by cBioportal.

**List Of Target Genes**			
PCGF5	DCP1A	ST3GAL5	MOGAT1
BMT2	ANG	RAD21	ZNF226
DYRK1A	PIK3R2	MTDH	CDC7
MSANTD4	PAWR	CHDH	KDELC2
ZBTB18	PPP1R12A	OSBPL10	CLK4
GPSM2	AASDHPPT	HAS3	N4BP2
GINM1	XPO1	GLCE	PAPD5
PGM3	CAND1	NUDT3	PHTF2
NCOA3	TBPL1	PLAGL2	MCAM
TRIM23	DBT	AGO2	ZNF264
LEAP2	SIX4	MLXIP	MACF1
TNRC6A	ORC4	MIB1	PRKAR1A
SCD	YME1L1	EDRF1	SEC23B
ZNF200	KIF5B	UHRF1BP1	ZNF507
SERPINE1	KCTD12	MYLIP	FOXA1
BLOC1S6	MFSD13A	ENPP4	LIMS1
RAB10	RACGAP1	ZSCAN29	KLF10
C4orf3	KIAA1143	ZNF85	INHBA
TFDP1	ZNF320	ZNF460	ITGB1
SP4	ELOVL5	COPS7A	TIMP3
BRAF			

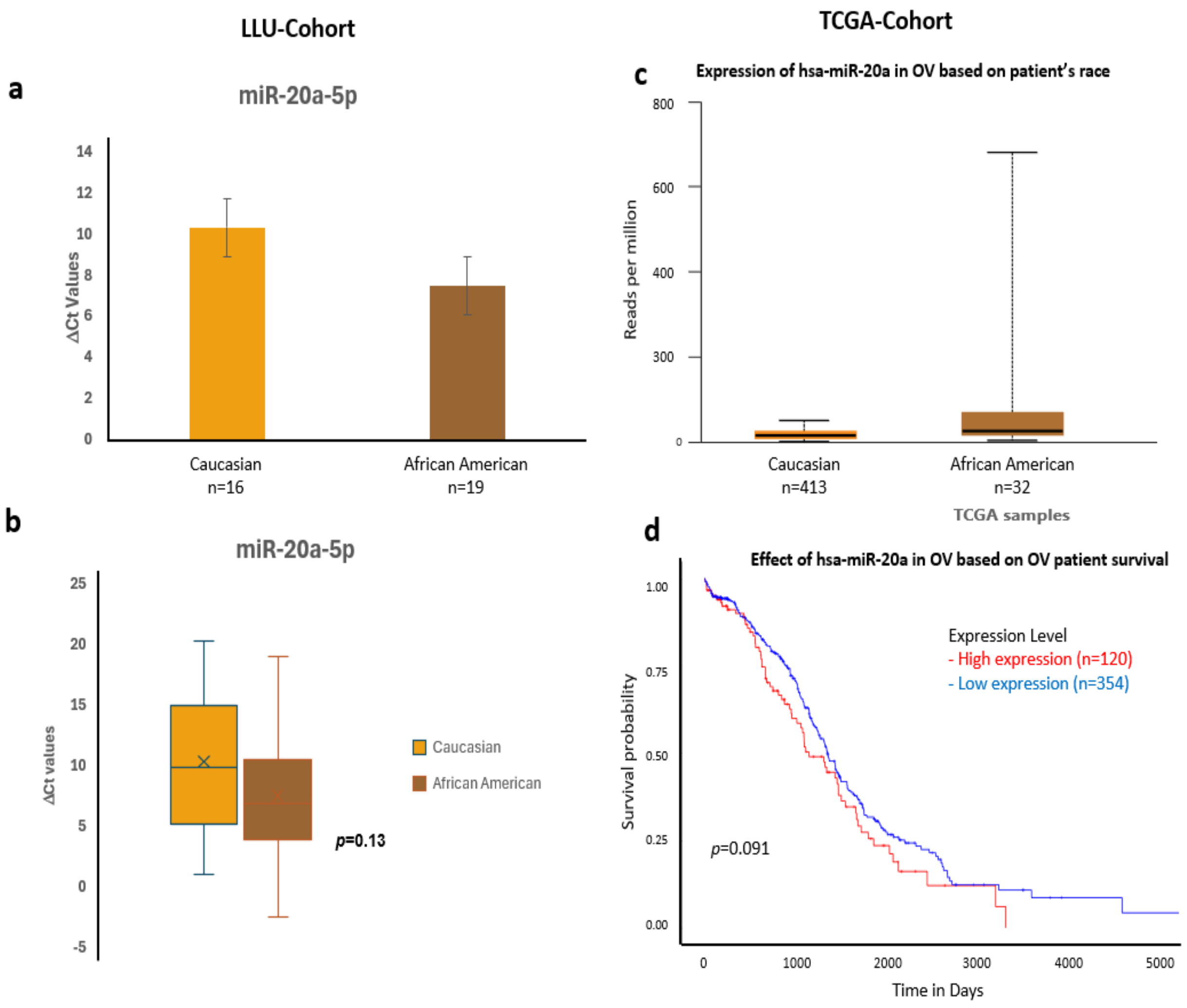

Figure S3. Comparative expression and survival analysis of miR-20a-5p in ovarian carcinoma across LLU and TCGA cohorts. (a-d) miR-20a-5p: Panels (a–b) show LLU cohort expression data comparing Caucasian and African American patients (ns, $p = 0.13$), with TCGA validation in panel (c), which also demonstrates relatively higher miR-20a-5p expression in African American samples. The TCGA survival analysis (d) indicates that elevated miR-20a-5p expression is associated with decreased overall survival (*ns*, $p = 0.091$). ns, not significant. Error bars represent mean ± SEM. Statistical analyses were performed using Student's t-test or log-rank test where appropriate.

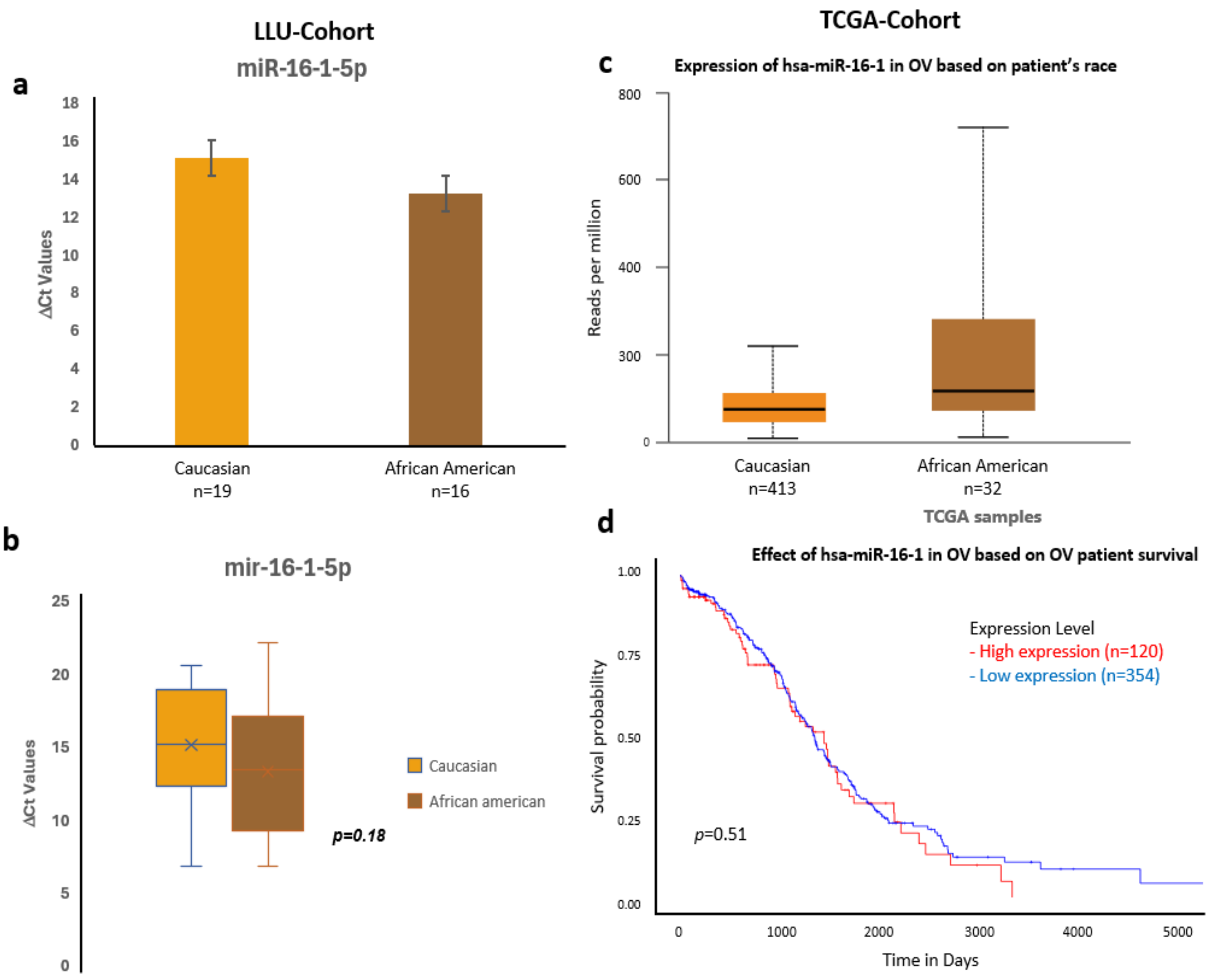

Figure S4. Comparative expression and survival analysis of miR-16-1-5p in ovarian carcinoma across LLU and TCGA cohorts. (a-d) miR-16-1-5p**:** Panels (a–b) show LLU cohort expression data comparing Caucasian and African American patients (ns, $p = 0.18$), with TCGA validation in panel (c), which also demonstrates relatively higher miR-16-1-5p expression in African American samples. The TCGA survival analysis (d) indicates that elevated miR-16-1-5p expression is associated with decreased overall survival (*ns*, $p = 0.51$). ns, not significant. Error bars represent mean ± SEM. Statistical analyses were performed using Student's t-test or log-rank test where appropriate.
